# Association of General Cognitive Functions with Gaming Use in Young Adults: A Comparison among Excessive Gamers, Regular Gamers and Non-Gamers

**DOI:** 10.3390/jcm10112293

**Published:** 2021-05-25

**Authors:** Joon Hwan Jang, Sun Ju Chung, Aruem Choi, Ji Yoon Lee, Bomi Kim, Minkyung Park, Susan Park, Jung-Seok Choi

**Affiliations:** 1 Department of Psychiatry, Seoul National University Health Service Center, Seoul 08826, Korea; jhjang602@naver.com (J.H.J.); alexa0479@naver.com (S.P.); 2 Department of Human Systems Medicine, Seoul National University College of Medicine, Seoul 03080, Korea; 3 Department of Psychiatry, SMG-SNU Boramae Medical Center, Seoul 07061, Korea; sunjujung1991@gmail.com (S.J.C.); choiar90@gmail.com (A.C.); idiyuni91@gmail.com (J.Y.L.); dreamykim@gmail.com (B.K.); reneedrv@gmail.com (M.P.); 4 Department of Psychiatry and Behavioral Science, Seoul National University College of Medicine, Seoul 03080, Korea

**Keywords:** cognitive function, intelligence, internet gaming disorder, gamers

## Abstract

This study aimed to examine the relationship of general cognitive function with gaming use, and to identify elements of intelligence predicting increased gaming use. In total, 160 young adults participated in this study. Two clinical groups (*n* = 97) were defined: excessive gaming users diagnosed with internet gaming disorder (IGD) (*n* = 64) and the high-risk users (*n* = 33). The control group (*n* = 63) was also divided into regular gamers (*n* = 14) and non-gamers (*n* = 49). Participants completed the Wechsler Adult Intelligence Scale-IV and self-reported questionnaires regarding IGD severity and gaming hours. The IGD group had significantly lower Full Scale Intelligence Quotient (FSIQ), Verbal Comprehension Index (VCI), and Processing Speed Index (PSI) scores, compared with regular gamers and non-gamers. The IGD group also exhibited lower Working Memory Index (WMI) scores, compared with non-gamers. The high-risk group demonstrated significantly lower PSI score, compared with non-gamers. Furthermore FSIQ, VCI, WMI, and PSI scores were significant predictors of gaming hours in the IGD group. For the high-risk group, FSIQ, WMI, and VCI scores were negatively associated with gaming hours. Our study demonstrates the need to address the importance of enhancing working memory and verbal ability, thus, preventing the development of gaming addiction among individuals at high-risk gamers.

## 1. Introduction

The frequency of internet gaming has increased with the global spread of internet use. In particular, online gaming and related activities have increased during the coronavirus (COVID-19) pandemic [1]. Internet gaming disorder (IGD) is regarded as a behavioral addiction, which means that gaming behavior is difficult to control and leads to difficulties in various domains. It is important to acknowledge that a significant increase in gaming may pose risks for vulnerable individuals. Among several factors related to IGD, cognitive impairment has been associated with behavioral addictions, such as IGD [2]. Previous studies have reported general cognitive deficits represented by impaired intelligence in several addictive disorders [3,4,5]. These findings might be explained by the association between intelligence and difficulties with self-regulation, as reflected in addictive behaviors. For example, children with intellectual disabilities showed a lack of self-regulation, compared with their normally developing peers [6]. Similarly, a study regarding the relationship between delay discounting and intelligence revealed that higher intelligence was associated with lower delay discounting, reflecting a higher level of self-control [7].

Among the various components of intelligence, verbal intelligence and working memory are known to be strongly associated with executive function, a frontal lobe function that is responsible for inhibition and self-control [8,9]. Previous researchers have examined the relationships among self-regulatory skills, verbal intelligence, and working memory. Ayduk et al. [10] found a significant association between self-regulation and verbal intelligence, which suggested that verbal intelligence was related to lower aggression among individuals who showed effective self-control skills. Petersen et al. [11] proposed that later self-regulation was predicted by verbal ability; weaker self-regulation skills mediated the effect of language deficits on subsequent behavior problems. In terms of working memory, Dučić [12] observed deficits in both working memory and self-control among individuals with intellectual disabilities. Furthermore, a reduced intellectual level was reportedly associated with more serious deficits in the development of self-control [13] and working memory capacity [14]. Previous research regarding the relationship between impulsive decision making and working memory found that during a delayed discounting task, the preference for a smaller immediate payoff over a larger delayed payoff was enhanced when the working memory load was higher. These findings suggested that limitations of working memory capacity may increase the likelihood of impulsive decision making [15].

Considering these findings, both verbal intelligence and working memory may be associated with self-control; deficits in these functions may lead to impaired self-regulation, which might increase the risk of addictive behaviors. In studies of individuals with alcohol use disorder, poor self-regulation was associated with executive function deficits, including lower intelligence; indeed, lower intelligence may be an important risk factor for problematic alcohol consumption [16,17,18]. However, there have been few direct studies of the relationship between intelligence deficits and excessive gaming use. An exception is the concept of “emotional intelligence”, which has been discussed in some addiction studies. However, it remains controversial whether this concept can objectively represent cognitive function, partially due to questions regarding its validity [19]. Notably, few studies have explored whether adult intelligence is related to self-control; most previous studies concerning this topic have assessed preschool, early childhood, or adolescent populations. Furthermore, it is difficult to ascertain whether lower intelligence is present in high-risk individuals who play games, but not at the level of IGD, and whether there is an intelligence difference between healthy controls who do not play any games and healthy controls who play games regularly. Such an approach could provide new insight into the relationship among general cognitive function, the degree of gaming use and impairment in self-regulation for IGD.

Therefore, this study aimed to investigate differences in general cognitive functioning according to the severity of gaming use and identify significant factors among intelligence indices that predict the extent of gaming use, with a focus on verbal intelligence and working memory. Based on the findings of prior studies [10,11,12], we hypothesized that general cognitive function would be negatively associated with the degree of gaming use, and that verbal ability and working memory would be significant predictors of the time devoted to gaming.

## 2. Materials and Methods

### 2.1. Participants

In total, 160 young adults (mean age: 24.60; 131 males, 29 females) participated in the study and were divided into three groups (IGD, high-risk, and healthy control), based on criteria in the Diagnostic and Statistical Manual of Mental Disorders Fifth Edition (DSM-5) [20]. The IGD group included individuals who met the IGD criteria in DSM-5. The high-risk group included individuals who played online games excessively, but not at the level of IGD; specifically, they met one to four of the nine DSM-5 criteria for IGD. The healthy controls met none of the IGD criteria. The healthy controls were further divided into regular gamers who regularly played online games, but did not meet any IGD criteria and non-gamers who did not play games. Patients in the IGD and high-risk groups were recruited in the SMG-SNU Boramae Medical Center in Seoul, Republic of Korea. All individuals with IGD or high-risk group were seeking treatment for problems related to excessive internet gaming and a clinically experienced psychiatrist diagnosed them with IGD or a high-risk status. The healthy controls were recruited using leaflets in the local community and posting promotional materials on the community websites; they were matched for age and sex with the IGD or high-risk groups. Individuals with intelligence levels below 70 or psychotic disorders, as well as individuals with Alcohol Use Disorder Identification Test scores above the cutoff, were excluded from the study.

### 2.2. Measures

Self-reporting questionnaires regarding demographic information, such as sex, age, education level, and time devoted to gaming were administered to all participants at addiction clinic of SMG-SNU Boramae Medical Center by a psychiatrist.

#### 2.2.1. DSM-5 Diagnostic Criteria for IGD

The criteria for IGD in the DSM-5 were used to classify participants into groups. These criteria comprise nine items: Persistent excessive use, tolerance, preoccupation, withdrawal, lack of other interests, functional impairment, deception of other people regarding game use, unsuccessful attempts to control internet use and escape. Individuals who met five or more of these items and who had experienced problems with daily life due to game use were diagnosed with IGD [20].

#### 2.2.2. Korean-Wechsler Adult Intelligence Scale-IV (K-WAIS-IV)

General cognitive functioning was measured using the K-WAIS-IV, which is the most frequently used and validated measure of the intelligence quotient (IQ) in Korean individuals aged 16 to 69 years. The K-WAIS-IV includes 15 subtests comprising the Full-scale IQ (FSIQ) and four indices, including the Verbal Comprehension Index (VCI), Perceptual Reasoning Index (PRI), Working Memory Index (WMI), and Processing Speed Index (PSI) [21]. The FSIQ score reflects the overall level of the individual’s intellectual functioning. The VCI measures verbal ability including verbal comprehension, reasoning, and conceptualization; the PRI assesses abilities in nonverbal conceptual reasoning, visual organization, and perception; the WMI reflects the ability to concentrate and working memory capacity; and the PSI represents psychomotor speed in response to nonverbal, visual stimuli [22].

#### 2.2.3. Young’s Internet Addiction test (Y-IAT)

The Y-IAT measures the level of internet addiction based on 20 items using five-point Likert scales. In this study, a modified Y-IAT made for assessing Internet games was used [23,24]. Scores range from 20 to 100; higher scores indicate higher levels of Internet addiction [25]. The Cronbach’s α coefficient for this scale was 0.964.

### 2.3. Measures Statistical Analyses

The chi-squared test and Kruskal-Wallis test were used to compare demographic characteristics and intelligence levels between groups. Kruskal-Wallis test was followed by Bonferroni’s test. Pearson’s correlation was calculated to identify relationships among intelligence indices, gaming hours, and severity of IGD, as measured by the Y-IAT. A simple linear regression analysis was conducted to examine whether the cognitive function indices as independent variables could predict gaming hours in total participants as well as in each group. IBM SPSS Statistics, version 21.0 (IBM Corp., Armonk, NY, USA) was used for all analyses.

## 3. Results

### 3.1. Demographic Information

Table 1 shows the participants’ demographic information among the IGD group, high-risk group and healthy control group. There were no significant differences in sex and age among groups, although the education level was significantly higher in the healthy controls than in the IGD group. Furthermore, gaming hours and Y-IAT scores significantly differed among groups. The IGD group devoted more time to online gaming and showed higher Y-IAT scores, compared with the other groups. Furthermore, the high-risk group significantly differed from the healthy controls with respect to the same variables.

Table 2 shows the demographic information for the four groups including the IGD group, high-risk group, regular gamers and non-gamers. There were no significant differences in sex or age. Significant differences were found in terms of education level, but post hoc analysis revealed no clear distinctions among the groups. In terms of gaming hours, the time devoted to gaming was significantly greater among individuals in the IGD group than among individuals in the other three groups. However, the difference between the high-risk group and regular gamers was not statistically significant. On the Y-IAT, the IGD group scored significantly higher, compared with the other groups.

### 3.2. Comparisons of General Cognitive Function Levels among Groups

Comparisons of intelligence levels among the IGD, high-risk, and healthy control groups are shown in the Table 3. The IGD and high-risk groups showed significantly lower FSIQ, VCI, and PSI scores, compared with the healthy controls.

The Table 4 shows a comparison of the intelligence levels of the four groups, indicating significant differences in FSIQ, VCI, WMI, and PSI scores. The IGD group showed significantly lower cognitive function indices, compared with regular gamers and non-gamers, on the FSIQ, VCI, and PSI indices. The high-risk group significantly differed from non-gamers, but not from regular gamers, in terms of the PSI score. WMI scores significantly differed between individuals with IGD and non-gamers; there were no group differences in PRI scores.

### 3.3. Correlation Coefficients

The results of correlational analyses regarding intelligence, gaming hours, and Y-IAT among all participants are shown in Table 5. The gaming hours was significantly negatively correlated with all cognitive function indices. The correlation between gaming hours and cognitive function indices was maintained after controlling for Y-IAT score (FSIQ: r = −0.430, *p* < 0.001; VCI: r = −0.432, *p* < 0.001; PRI: r = −0.205, *p* = 0.010; WMI: r = −0.337, *p* < 0.001; PSI: r = −0.343, *p* < 0.001). Y-IAT scores were positively correlated with gaming hours but were not significantly correlated with any intelligence indices, with the exception of the PSI.

### 3.4. Cognitive Function Indices Predicting the Extent of Gaming Use

The results of regression analyses are shown in Table 6. In the overall sample, the analyses were conducted after controlling for education, due to group differences in demographic information, and Y-IAT score. Across participants, various cognitive function indices were significant predictors of gaming hours, suggesting that they were associated with the quantitative aspects of excessive gaming. In analyses of separate groups, FSIQ (*β* = −0.346, *p* = 0.007), VCI (*β* = −0.386, *p* =0.003), WMI (*β* = −0.265, *p* = 0.032), and PSI (*β* = −0.263, *p* = 0.035) were negatively associated with gaming hours in the IGD group. In the high-risk group, FSIQ (*β* = −0.481, *p* = 0.046) and WMI (*β* = −0.489, *p* = 0.038) were significant predictors of the time devoted to gaming. VCI (*β* = −0.459, *p* = 0.053) was also fairly significant predictors in the high-risk group.

## 4. Discussion

The present study examined the differences in general cognitive functioning, as measured by the K-WAIS-IV, based on the extent of gaming use. It determined the relationship between intelligence indices and gaming use in young adults. To the best of our knowledge, this is the first study to address the association of intelligence indices with the extent of gaming use in young adults.

The primary focus of the present study was to determine the relationship of differences in cognitive function with the extent of gaming use. As expected, both the IGD and high-risk groups showed generally lower cognitive function indices, compared with healthy controls. There were significant differences among the IGD, high-risk, and healthy control groups in FSIQ, VCI, and PSI scores. Furthermore, the IGD group exhibited significantly lower scores on the FSIQ, VCI, and PSI, compared with regular gamers and non-gamers; the high-risk group differed only from non-gamers on the PSI. In addition, the IGD group showed lower WMI scores, compared with the healthy controls, especially non-gamers.

Individuals with IGD exhibited marked reduction of general intellectual functioning and lower psychomotor speed, compared with healthy controls. The results regarding the comparison of intelligence between individuals with IGD and control groups have been inconsistent. Some studies have found significant differences between addicted gamers and healthy controls [26], while others showed no group differences [23,27]. However, in most previous studies, the estimated intelligence quotient has generally tended to be lower in participants with IGD than in healthy controls, although this difference may not be statistically significant. In this context, cognitive functioning may be relatively impoverished in patients with IGD, compared with healthy controls. Furthermore, impulsive individuals are more likely to show reduced performance in tests of processing speed [28], as they may easily become bored with the task or exhibit a lack of motivation. Considering that individuals with IGD tend to have high impulsivity [23], these characteristics of IGD might have been reflected in the present results. In addition, the high-risk group showed lower PSI scores, compared with non-gamers. This indicates that individuals at high risk of IGD exhibit reduced cognitive function, suggestive of early objective factors for increased risk of IGD. With respect to VCI, both the IGD and high-risk groups showed significantly lower scores, compared with healthy controls. Furthermore, VCI scores differed between individuals with IGD and healthy controls, regardless of whether the control group engaged in online gaming. Individuals who engaged in excessive gaming may lack the ability to comprehend or conceptualize verbal information.

WMI scores differed between patients with IGD and healthy controls. Furthermore, the comparison among all four groups revealed a more pronounced difference between patients with IGD and non-gamers. These results suggest that patients with IGD are more likely to experience difficulty in retaining and manipulating information, to have lower working memory capacity and to be more distractible. In a previous meta-analysis of 40 studies examining cognitive impairments associated with problematic internet use, affected individuals were found to exhibit significant deficits in working memory, inhibitory control, and decision making, compared with the control group [29]. In contrast to the other indices of cognitive function, there were no group differences in PRI scores. Therefpre, unlike other indicators, non-verbal reasoning and visual processing may not be related to the severity of IGD. Physical and mental activities involved in playing games may be associated with preserved visual perception processing in gamers.

The second focus of the present study was to identify factors among the intelligence indices that predicted the quantitative degree of excessive gaming use. The number of gaming hours was negatively associated with all cognitive function indices; all of the indices were significant predictors of the extent of gaming among the overall sample. These results indicate that individuals with lower cognitive function indices are more likely to spend time in online gaming; they indirectly suggest that they may have difficulty controlling addictive behaviors, such as excessive internet gaming. Previous studies have consistently found that general cognitive function is positively associated with self-regulation, supporting the results of this study [6,7]. Predictors of increased gaming hours in both the IGD and high-risk groups were FSIQ, WMI and VCI scores. This finding suggest that, as overall cognitive functioning decreases (e.g., verbal comprehension or working memory), the time devoted to playing games increases; this may enhance the risk of addiction. These results may be attributable to associations of working memory and verbal intelligence with self-regulatory functions. Previous studies have reported a significant interaction between self-regulation and verbal intelligence [10]; worse self-regulation was predicted by language deficits [11]. Furthermore, verbal ability was positively related to inhibitory control [30,31]. In terms of working memory, Behrouzmanesh et al. [32] found a significant association between working memory and self-regulatory behavior. Moreover, individuals with good working memory capacity tended to act in a more goal-directed and focused manner in situations demanding attention [33]. In contrast, individuals who were addicted to the internet had impaired working memory and executive function, compared with the control group [34]. Similarly, Nie et al. [35] reported impairments in working memory and response inhibition among individuals with internet addiction and individuals who had attention deficit hyperactivity disorder and internet addiction, especially in response to stimuli associated with the internet. In this context, deficits in verbal ability and working memory may reduce the ability to cope with circumstances that demand self-regulation skills, such as excessive gaming. Moreover, the increase in gaming hours among individuals who played games regularly, but did not meet any of the criteria for IGD, was more likely affected by working memory deficits. Notably, the high-risk group had features of the IGD group, including FSIQ and WMI as predictive factors. This suggests that the high-risk group had characteristics that could be affected by both verbal ability and working memory, perhaps because they were in a transitional period moving toward addiction, but they were neither in the IGD group nor regular gamers. It can pose a potential risk to high-risk individuals of developing problematic gaming activity patterns in response to the COVID-19 pandemic. Excessive engagement in gaming activity may contribute to functional impairment during prolonged quarantine periods. Therefore, it is essential to maintain a controlled level of gaming activity through a well-balanced daily routine [36].

The present study has some limitations. First, a causal relationship between intelligence indices and gaming hours could not be identified, as the analyses were based on a cross-sectional design. Therefore, future research should address this issue using a longitudinal design. Second, it is difficult to generalize these findings to other populations because the sample used in this study consisted of a small number of young adults. Moreover, there were more male participants than female participants. Therefore, more diverse and larger samples are needed in further research. Third, mood status can influence associations between intelligence and the extent of gaming, especially among patients with IGD and individuals at high-risk for IGD. However, this study could not investigate depressive or anxiety symptoms.

## 5. Conclusions

Despite these limitations, we found relationships between cognitive function and the extent of gaming activity. These results suggest that individuals who engage in excessive internet gaming tend to have lower cognitive function, especially in terms of verbal ability and working memory, which are associated with excessive gaming in individuals at high-risk. Our study demonstrates the need to address the importance of enhancing verbal ability and working memory, thus, preventing the development of internet gaming addiction among individuals at high-risk.

## Figures and Tables

**Table 1:** Comparison of demographics among three groups (*n* = 160).

Variables	IGD ^1^ (*n* = 64)	High-Risk ^2^ (*n* = 33)	Healthy Controls ^3^ (*n* = 63)	H/x^2^	*p* Value	Bonferroni ^4^
Gender (M/F)	58/6	26/7	47/16	5.759	0.056	
Age	24.45 (5.44)	25.52 (5.50)	24.27 (3.16)	1.452	0.484	
Education	13.23 (1.48)	13.55 (1.49)	14.35 (1.81)	17.332	<0.001	1 < 3
Gaming hours	5.12 (3.80)	2.08 (2.07)	0.33 (0.74)	92.735	<0.001	1 > 2 > 3
Y-IAT	61.17 (16.95)	38.73 (12.38)	30.25 (8.69)	86.542	<0.001	1 > 2 > 3

^1^ Values are expressed as means (standard deviations). ^2^ Values are expressed as means (standard deviations). ^3^ Values are expressed as means (standard deviations). ^4^ 1 = IGD; 2 = high-risk group; 3 = healthy controls. IGD, internet gaming disorder; Y-IAT, Young’s Internet Addiction Test.

**Table 2:** Comparison of demographics among four groups (*n* = 160).

Variables	IGD ^1^ (*n* = 64)	High-Risk ^2^ (*n* = 33)	Regular Gamer ^3^ (*n* = 14)	Non-Gamer ^4^ (*n* = 49)	H/x^2^	*p* Value	Bonferroni ^5^
Gender (M/F)	58/6	26/7	12/2	35/14	7.256	0.065	
Age	24.45 (5.44)	25.52 (5.50)	23.43 (2.88)	24.51 (3.22)	1.795	0.616	
Education	13.23 (1.48)	13.55 (1.49)	14.29 (1.86)	14.37 (1.81)	17.352	<0.001	
Gaming hours	5.12 (3.80)	2.08 (2.07)	1.50 (0.85)	0.00 (0.00)	104.728	<0.001	1 > 2, 3 > 4
Y-IAT	61.17 (16.95)	38.73 (12.38)	28.50 (5.11)	30.76 (9.45)	86.731	<0.001	1 > 2, 3, 4

^1^ Values are expressed as means (standard deviations). ^2^ Values are expressed as means (standard deviations). ^3^ Values are expressed as means (standard deviations). ^4^ Values are expressed as means (standard deviations). ^5^ 1 = IGD; 2 = high-risk group; 3 = regular gamer; 4 = non-gamer. IGD, internet gaming disorder; Y-IAT, Young’s Internet Addiction Test.

**Table 3:** Comparison of intelligence level among three groups (*n* = 160).

Variables	IGD ^1^ (*n* = 64)	High-Risk ^2^ (*n* = 33)	Healthy Controls ^3^ (*n* = 63)	H/x^2^	*p* Value	Bonferroni ^4^
FSIQ	102.33 (16.47)	107.12 (14.73)	115.35 (10.87)	21.576	<0.001	1,2 < 3
VCI	104.19 (14.52)	107.58 (15.47)	115.70 (11.71)	19.179	<0.001	1, 2 < 3
PRI	106.27 (15.73)	107.33 (14.84)	109.52 (15.34)	1.724	0.422	
WMI	104.92 (16.92)	108.70 (13.62)	114.40 (13.07)	10.331	0.006	1 < 3
PSI	93.14 (16.08)	99.79 (14.00)	109.67 (13.95)	35.174	<0.001	1, 2 < 3

^1^ Values are expressed as means (standard deviations). ^2^ Values are expressed as means (standard deviations). ^3^ Values are expressed as means (standard deviations). ^4^ 1 = IGD; 2 = high-risk group; 3 = healthy controls. IGD, internet gaming disorder; FSIQ, Full Scale Intellectual Quotient; VCI, Verbal Comprehension Index; PRI, Perceptual Reasoning Index; WMI, Working Memory Index; PSI, Processing Speed Index.

**Table 4:** Comparison of intelligence level among four groups (*n* = 160).

Variables	IGD ^1^ (*n* = 64)	High-Risk ^2^ (*n* = 33)	Regular Gamer ^3^ (*n* = 14)	Non-Gamer ^4^ (*n* = 49)	H/x^2^	*p* Value	Bonferroni ^5^
FSIQ	102.33 (16.47)	107.12 (14.73)	115.07 (9.49)	115.43 (11.32)	21.577	<0.001	1 < 3, 4
VCI	104.19 (14.52)	107.58 (15.47)	118.50 (8.46)	114.90 (12.45)	20.246	<0.001	1 < 3, 4
PRI	106.27 (15.73)	107.33 (14.84)	108.79 (15.67)	109.73 (15.41)	1.728	0.631	
WMI	104.92 (16.92)	108.70 (13.62)	113.57 (16.08)	114.63 (12.26)	10.417	0.015	1 < 4
PSI	93.14 (16.08)	99.79 (14.00)	107.07 (9.92)	110.41 (14.90)	35.345	<0.001	1 < 3, 4 2 < 4

^1^ Values are expressed as means (standard deviations). ^2^ Values are expressed as means (standard deviations). ^3^ Values are expressed as means (standard deviations). ^4^ Values are expressed as means (standard deviations). ^5^ 1 = IGD; 2 = high-risk group; 3 = regular gamer; 4 = non-gamer. IGD, internet gaming disorder; FSIQ, Full Scale Intellectual Quotient; VCI, Verbal Comprehension Index; PRI, Perceptual Reasoning Index; WMI, Working Memory Index; PSI, Processing Speed Index.

**Table 5:** Correlations of variables in total sample (*n* = 160).

	1	2	3	4	5	6	7
1. VCI	1						
2. PRI	0.375 ***	1					
3. WMI	0.513 ***	0.365 ***	1				
4. PSI	0.452 ***	0.378 ***	0.448 ***	1			
5. FSIQ	0.083 ***	0.721 ***	0.700 ***	0.737 ***	1		
6. Gaming hours	−0.411 ***	−0.173 *	−0.315 ***	−0.408 ***	−0.437 ***	1	
7. Y-IAT	−0.098	−0.010	−0.076	−0.242 **	−0.154	0.561 ***	1
M	109.42	107.77	108.76	101.02	108.44	2.64 ^1^	44.13
SD	14.58	15.37	17.14	16.51	15.22	3.40 ^1^	19.56

* *p* < 0.05. ** *p* < 0.01. *** *p* < 0.001. ^1^ Time (hours). VCI, Verbal Comprehension Index; PRI, Perceptual Reasoning Index; WMI, Working Memory Index; PSI, Processing Speed Index; FSIQ, Full Scale Intellectual Quotient; M = mean; SD = Standard deviation.

**Table 6:** Simple linear regression of intelligence index on gaming hours.

Group	Independent Variables	Gaming Hours						*p* Value
		*B*	*SE*	*β*	*t*	*R^2^*	*F*	
Total participants (*n* = 160)	FSIQ	−0.075	0.013	−0.337	−5.562	0.458	43.974 ***	<0.001
	VCI	−0.078	0.014	−0.336	−5.522	0.457	43.747 ***	<0.001
	PRI	−0.035	0.014	−0.158	−2.491	0.376	31.275 ***	<0.001
	WMI	−0.058	0.014	−0.264	−4.268	0.419	37.441 ***	<0.001
	PSI	−0.055	0.013	−0.270	−4.245	0.418	37.342 ***	<0.001
IGD (*n* = 64)	FSIQ	−0.080	0.027	−0.346	−2.934	0.183	4.492 **	0.007
	VCI	−0.101	0.030	−0.386	−3.315	0.211	5.340 **	0.003
	PRI	−0.048	0.030	−0.199	−1.617	0.105	2.352	0.081
	WMI	−0.060	0.027	−0.265	−2.200	0.136	3.146 *	0.032
	PSI	−0.062	0.029	−0.263	−2.141	0.132	3.055 *	0.035
High risk group (*n* = 33)	FSIQ	−0.068	0.026	−0.481	−2.633	0.238	3.014 *	0.046
	VCI	−0.061	0.024	−0.459	−2.557	0.229	2.875	0.053
	PRI	−0.048	0.028	−0.341	−1.685	0.140	1.569	0.218
	WMI	−0.074	0.027	−0.489	−2.729	0.248	3.196 *	0.038
	PSI	−0.043	0.026	−0.289	−1.656	0.137	1.535	0.226
Regular gamer (*n* = 14)	FSIQ	−0.043	0.025	−0.475	−1.703	0.297	1.407	0.297
	VCI	−0.078	0.045	−0.769	−1.719	0.300	1.427	0.292
	PRI	−0.015	0.016	−0.278	−0.954	0.169	0.676	0.586
	WMI	−0.011	0.016	−0.210	−0.683	0.133	0.513	0.682
	PSI	−0.019	0.026	−0.218	−0.737	0.140	0.541	0.665

* *p* < 0.05. ** *p* < 0.01. *** *p* < 0.001. FSIQ, Full Scale Intellectual Quotient; VCI, Verbal Comprehension Index; PRI, Perceptual Reasoning Index; WMI, Working Memory Index; PSI, Processing Speed Index; IGD, Internet gaming disorder.

## Data Availability

The data presented in this study are available on request from the corresponding author.
